# Correction: Łyczko, J. et al. HS-SPME Analysis of True Lavender (*Lavandula angustifolia* Mill.) Leaves Treated by Various Drying Methods. *Molecules* 2019, *24*, 764

**DOI:** 10.3390/molecules24244538

**Published:** 2019-12-11

**Authors:** Jacek Łyczko, Klaudiusz Jałoszyński, Mariusz Surma, Klaudia Masztalerz, Antoni Szumny

**Affiliations:** 1 Faculty of Biotechnology and Food Science, Wrocław University of Environmental and Life Sciences, Norwida 25, 50-375 Wrocław, Poland; antoni.szumny@upwr.edu.pl; 2 Institute of Agricultural Engineering, Wrocław University of Environmental and Life Sciences, Chełmońskiego 37-41, 51-630 Wrocław, Poland; klaudiusz.jaloszynski@upwr.edu.pl (K.J.); mariusz.surma@upwr.edu.pl (M.S.); klaudia.urbanska@upwr.edu.pl (K.M.)

The author wishes to make the following corrections to this paper [1]. Due to a format mistake, we would like to replace:

**Table 3:** Variability of major volatile constituents, linalool, and total essential oil of true lavender leaves caused by various drying methods.

Compound		Drying Method						
	Fresh	CD 50 °C	CD 60 °C	CD 70 °C	CPD-VMFD	VMD 240 W	VMD 360 W	VMD 480 W
	Content [%] ^1^							
*p*-Cymene	2.58 ^a^	2.73^c^	2.72 ^c^	1.76 ^d^	2.01 ^de^	2.15 ^e^	2.27 ^e^	3.50 ^b^
*o*-Cymene	4.81 ^a^	6.26 ^c^	5.65 ^d^	3.05 ^f^	3.69 ^e^	4.62 ^g^	4.73 ^g^	8.08 ^b^
Limonene	3.42 ^a^	3.27 ^f^	3.47 ^f^	1.31 ^e^	3.08 ^cf^	1.97 ^de^	2.41 ^cd^	6.99 ^b^
Eucalyptol	7.28 ^a^	5.01 ^bc^	3.71 ^d^	5.12 ^b^	3.98 ^cd^	3.25 ^d^	3.74 ^d^	3.44 ^d^
1-Octen-3-ol acetate	3.80 ^a^	2.70 ^de^	2.82 ^de^	4.23 ^c^	6.22 ^b^	2.10 ^e^	4.42 ^c^	3.68 ^cd^
Camphor	2.09 ^a^	2.32 ^b^	1.40 ^d^	1.89 ^c^	0.40 ^f^	1.12 ^e^	0.34 ^f^	0.39 ^f^
Borneol + Lavandulol	4.66 ^a^	7.63 ^b^	5.75 ^d^	6.07 ^d^	1.37 ^e^	4.78 ^c^	1.46 ^e^	1.35 ^e^
*m*-Cymen-8-ol	2.09 ^a^	3.18 ^c^	2.48 ^d^	3.67 ^b^	0.02 ^e^	2.74 ^d^	0.08 ^e^	0.07 ^e^
*p*-Cymen-8-ol	4.09 ^a^	6.31 ^c^	6.05 ^c^	7.17 ^b^	1.10 ^d^	6.07 ^c^	1.04 ^d^	0.89 ^d^
Cumin aldehyde	1.92 ^a^	3.59 ^c^	3.74 ^c^	4.48 ^b^	0.59 ^d^	4.30 ^b^	0.66 ^d^	0.59 ^d^
Linalyl acetate	2.21 ^a^	3.46 ^d^	11.06 ^b^	4.23 ^d^	1.60 ^e^	5.29 ^c^	1.66 ^e^	1.75 ^e^
Bornyl acetate	5.57 ^a^	3.54 ^c^	2.36 ^e^	4.04 ^b^	0.07 ^f^	3.07 ^d^	0.07 ^f^	0.14 ^f^
(*E*)-Caryophyllene	6.11 ^a^	2.11 ^d^	2.78 ^c^	3.51 ^f^	6.38 ^b^	4.85 ^e^	5.28 ^e^	3.98 ^f^
γ-Cadinene	10.53 ^a^	3.67 ^f^	4.43 ^ef^	4.74 ^e^	8.48 ^cd^	7.80 ^d^	9.20 ^c^	5.88 ^b^
Caryophyllene oxide	3.31 ^a^	2.43 ^c^	1.63 ^b^	2.12 ^bc^	2.24 ^bc^	2.11 ^bc^	2.47 ^c^	1.89 ^bc^
τ-Cadinol	2.04 ^a^	1.62 ^d^	1.44 ^d^	2.78 ^c^	2.74 ^c^	3.37 ^bc^	3.85 ^b^	1.74 ^d^
Ʃ-Linalool TOTAL essential oil [mL 100g^−1^ dw] ^2^	66.51 ^a^ 0.38 ^a^ 3.082 ^a^	59.83 ^b^ 4.32 ^c^ 0.588 ^e^	61.42 ^b^ 6.33 ^b^ 0.726 ^f^	60.17 ^b^ 4.71 ^c^ 0.992 ^cd^	43.97 ^c^ 0.76 ^ad^ 0.921 ^bc^	59.59 ^b^ 6.62 ^b^ 1.075 ^d^	43.68 ^c^ 0.73 ^ad^ 0.881 ^b^	42.47 ^c^ 1.16 ^d^ 1.302 ^g^

^1^ Values followed by the same letter within a row are not significantly different (*p* > 0.05, Duncan’s test); ^2^ Values obtained from steam distillation in Deryng apparatus.

with:

**Table 3:** Variability of major volatile constituents, linalool, and total essential oil of true lavender leaves caused by various drying methods.

Compound		Drying Method						
	Fresh	CD 50 °C	CD 60 °C	CD 70 °C	CPD-VMFD	VMD 240 W	VMD 360 W	VMD 480 W
	Content [%] ^1^							
*p*-Cymene	2.58 ^a^	2.73 ^c^	2.72 ^c^	1.76 ^d^	2.01 ^d,e^	2.15 ^e^	2.27 ^e^	3.50 ^b^
*o*-Cymene	4.81 ^a^	6.26 ^c^	5.65 ^d^	3.05 ^f^	3.69 ^e^	4.62 ^g^	4.73 ^g^	8.08 ^b^
Limonene	3.42 ^a^	3.27 ^f^	3.47 ^f^	1.31 ^e^	3.08 ^c,f^	1.97 ^d,e^	2.41 ^c,d^	6.99 ^b^
Eucalyptol	7.28 ^a^	5.01 ^b,c^	3.71 ^d^	5.12 ^b^	3.98 ^c,d^	3.25 ^d^	3.74 ^d^	3.44 ^d^
1-Octen-3-ol. acetate	3.80 ^a^	2.70 ^d,e^	2.82 ^d,e^	4.23 ^c^	6.22 ^b^	2.10 ^e^	4.42 ^c^	3.68 ^c,d^
Camphor	2.09 ^a^	2.32 ^b^	1.40 ^d^	1.89 ^c^	0.40 ^f^	1.12 ^e^	0.34 ^f^	0.39 ^f^
Borneol + Lavandulol	4.66 ^a^	7.63 ^b^	5.75 ^d^	6.07 ^d^	1.37 ^e^	4.78 ^c^	1.46 ^e^	1.35 ^e^
*m*-Cymen-8-ol	2.09 ^a^	3.18 ^c^	2.48 ^d^	3.67 ^b^	0.02 ^e^	2.74 ^d^	0.08 ^e^	0.07 ^e^
*p*-Cymen-8-ol	4.09 ^a^	6.31 ^c^	6.05 ^c^	7.17 ^b^	1.10 ^d^	6.07 ^c^	1.04 ^d^	0.89 ^d^
Cumin aldehyde	1.92 ^a^	3.59 ^c^	3.74 ^c^	4.48 ^b^	0.59 ^d^	4.30 ^b^	0.66 ^d^	0.59 ^d^
Linalyl acetate	2.21 ^a^	3.46 ^d^	11.06 ^b^	4.23 ^d^	1.60 ^e^	5.29 ^c^	1.66 ^e^	1.75 ^e^
Bornyl acetate	5.57 ^a^	3.54 ^c^	2.36 ^e^	4.04 ^b^	0.07 ^f^	3.07 ^d^	0.07 ^f^	0.14 ^f^
Caryophyllene <(E)->	6.11 ^a^	2.11 ^d^	2.78 ^c^	3.51 ^f^	6.38 ^b^	4.85 ^e^	5.28 ^e^	3.98 ^f^
γ-Cadinene	10.53 ^a^	3.67 ^f^	4.43 ^ef^	4.74 ^e^	8.48 ^c,d^	7.80 ^d^	9.20 ^c^	5.88 ^b^
Caryophyllene oxide	3.31 ^a^	2.43 ^c^	1.63 ^b^	2.12 ^b,c^	2.24 ^b,c^	2.11 ^b,c^	2.47 ^c^	1.89 ^b,c^
τ-Cadinol	2.04 ^a^	1.62 ^d^	1.44 ^d^	2.78 ^c^	2.74 ^c^	3.37 ^b,c^	3.85 ^b^	1.74 ^d^
Ʃ	66.51 ^a^	59.83 ^b^	61.42 ^b^	60.17 ^b^	43.97 ^c^	59.59 ^b^	43.68 ^c^	42.47 ^c^
Linalool	0.38 ^a^	4.32 ^c^	6.33 ^b^	4.71 ^c^	0.76 ^a,d^	6.62 ^b^	0.73 ^a,d^	1.16 ^d^
TOTAL essential oil [mL 100g^−1^ dw] ^2^	3.082 ^a^	0.588 ^e^	0.726 ^f^	0.992 ^c,d^	0.921 ^b,c^	1.075 ^d^	0.881 ^b^	1.302 ^g^

^1^ Values followed by the same letter within a row are not significantly different (*p* > 0.05, Duncan’s test); ^2^ Values obtained from steam distillation in Deryng apparatus.

Moreover, in the published article [1], the authors realized there were some errors in the Funding section and thus wish to replace the Funding section with the paragraph below:

**Funding:** This research was funded by the Faculty of Biotechnology and Food Science, Wrocław University of Environmental and Life Sciences, grant number B030/0003/1 and under the program of the Minister of Science and Higher Education “Strategy of Excellence University of Research” in 2018–2019 project number 0019/SDU/2018/18 in the amount of PLN 700 000.

The authors would like to apologize for any inconvenience caused to the readers by the change. The change does not affect the scientific results. The manuscript will be updated, and the original will remain online on the article webpage, with a reference to this Correction. 

## References

[1] Łyczko J., Jałoszyński K., Surma M., Masztalerz K., Szumny A. (2019). HS-SPME Analysis of True Lavender (*Lavandula angustifolia* Mill.) Leaves Treated by Various Drying Methods. Molecules.

