# Supplementary material for: HS-SPME Analysis of True Lavender (Lavandula angustifolia Mill.) Leaves Treated by Various Drying Methods
Source: Molecules. 2019 Feb 20;24(4):764. doi: 10.3390/molecules24040764 (PMC6412978; doi:10.3390/molecules24040764)
Supplement: Supplementary file 1 [file molecules-24-00764-s001.zip › Readme.pdf]

„Supplementary materials" explanation:

All analyses were performed on Varian Saturn2000 and processed with using Varian MS Workstation and ACD/Spectrus Processor 2017.2.1. Obtained chromatograms are presented in file named "Chromatograms.pdf". They are presented in following order: 3 replicates of fresh samples, 3 replicates of CD 50ºC, 3 replicates of CD 60 ºC, 3 replicates of CD 70 ºC, 3 replicates of CPD-VMFD, 3 replicates of VMD 240 W, 3 replicates of VMD 360 W, 3 replicates of VMD 480 W.

Moreover, mass spectra for unidentified compounds are presented in file named "unknown_compounds_mass_spectra.pdf" in order corresponding to Table 2.

Tables 1, 2 and 3 are included in Excel file named "Tables.xlsx".

Overall, following files have been submitted as supplementary materials in zipped folder "supplementary_materials.zip":

- Readme.pdf
- Chromatograms.pdf
- Unknown_compounds_mass_spectra.pdf
- Tables.xlsx
