# Supplementary figures and images for: HS-SPME Analysis of True Lavender (Lavandula angustifolia Mill.) Leaves Treated by Various Drying Methods
Source: Molecules. 2019 Feb 20;24(4):764. doi: 10.3390/molecules24040764 (PMC6412978; doi:10.3390/molecules24040764)

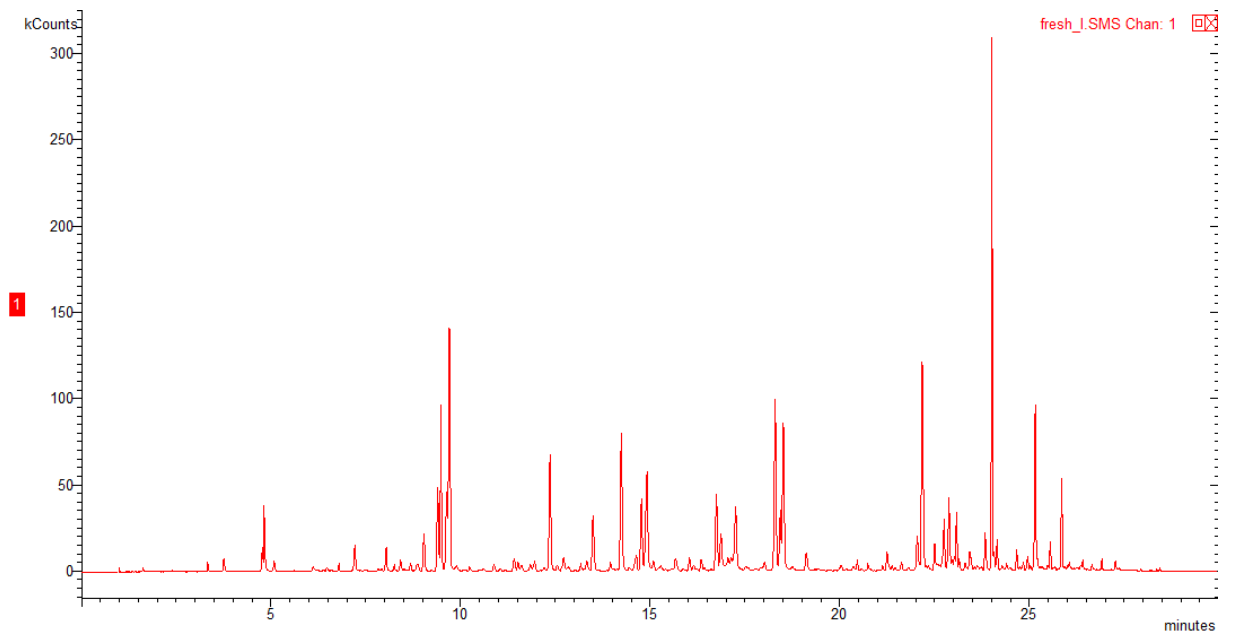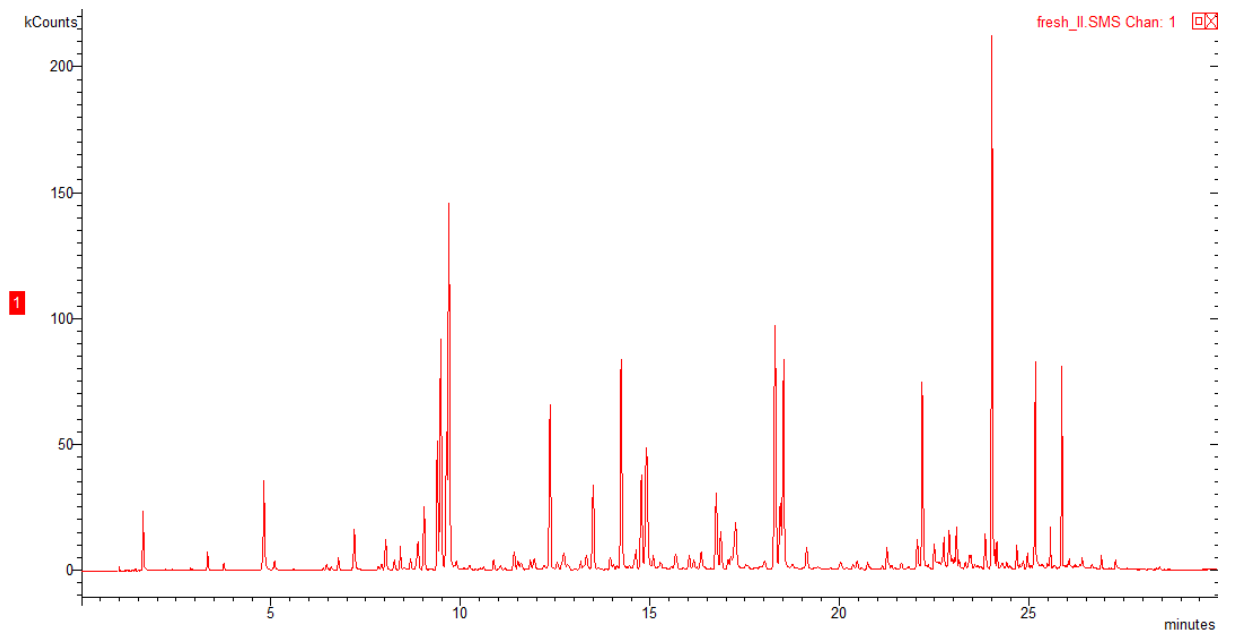

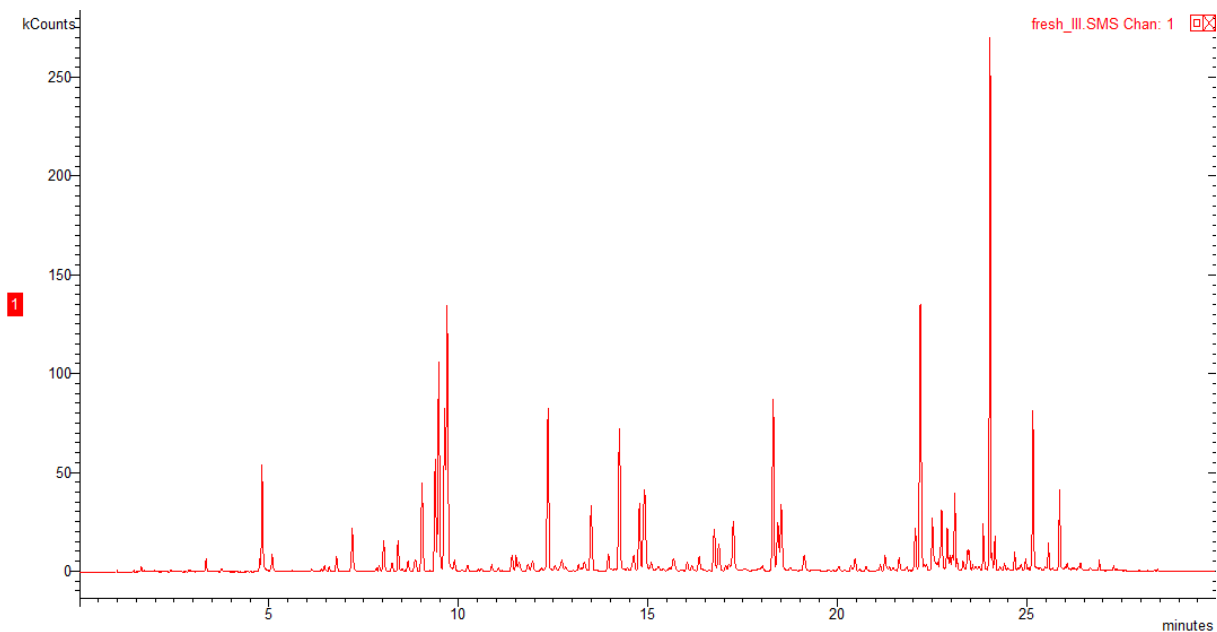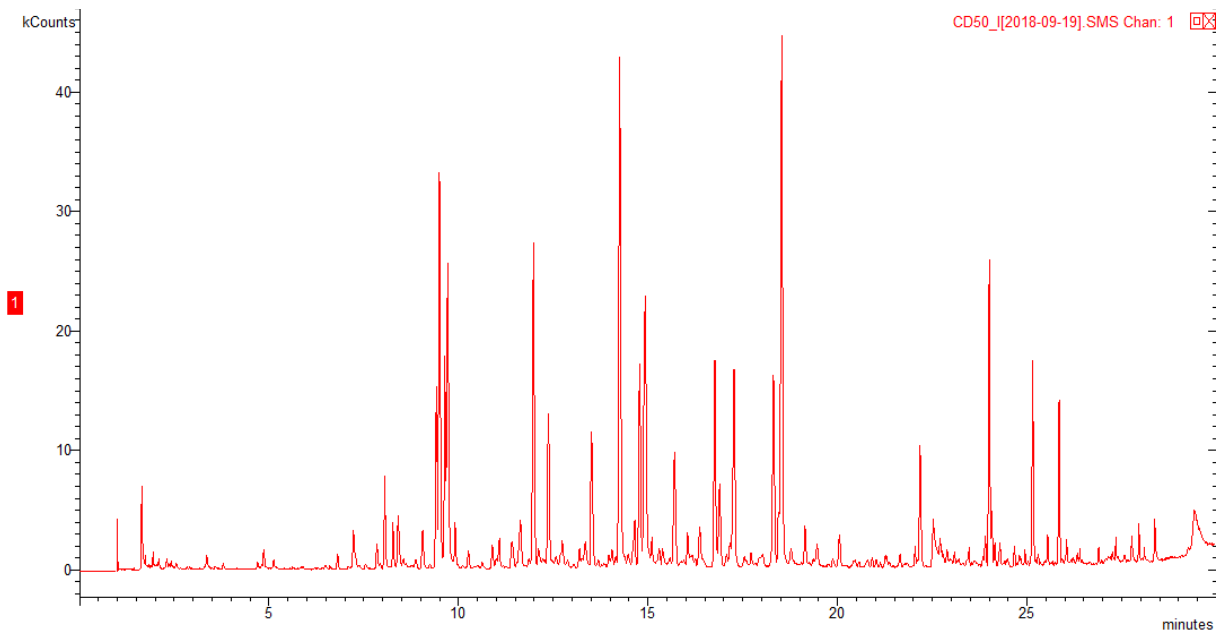

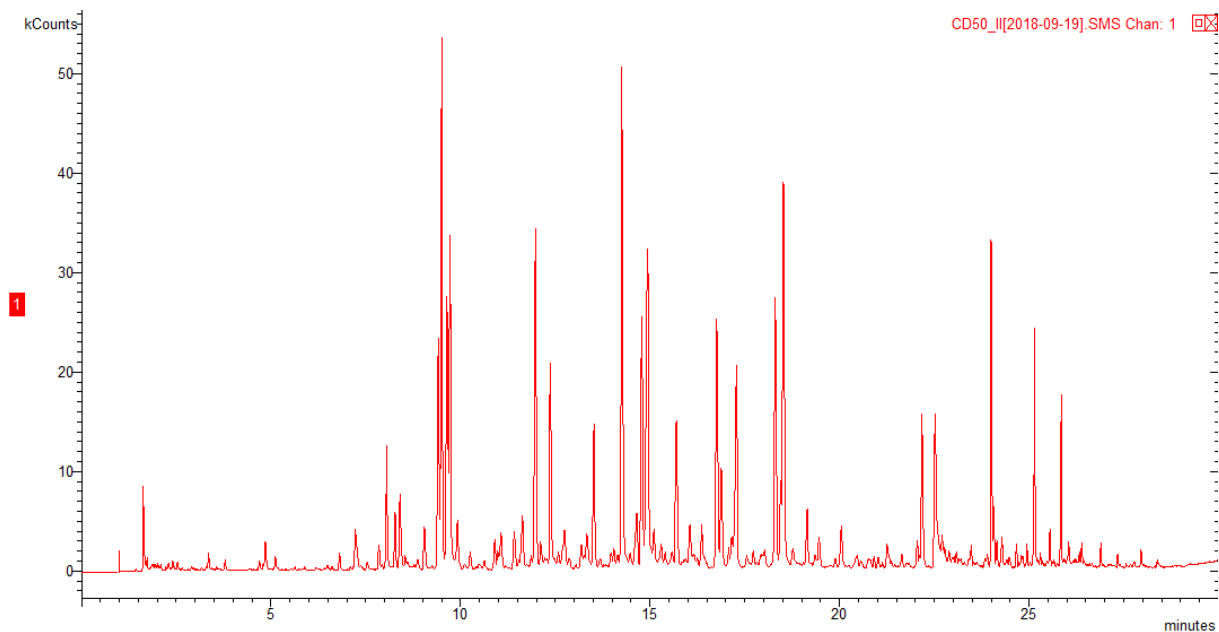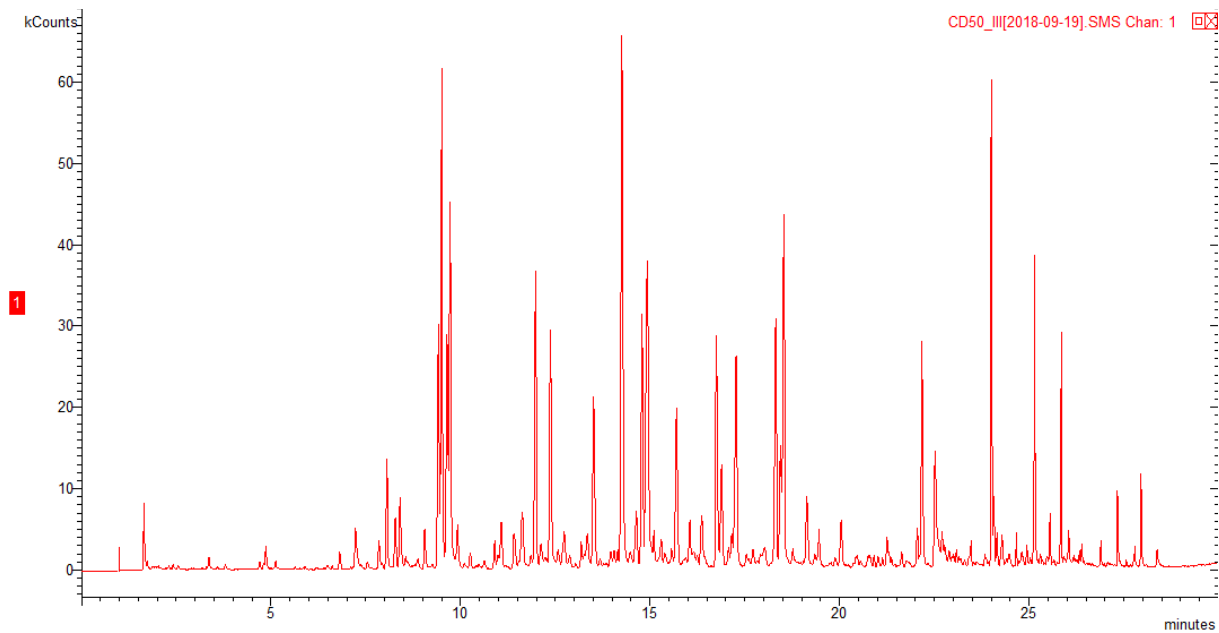

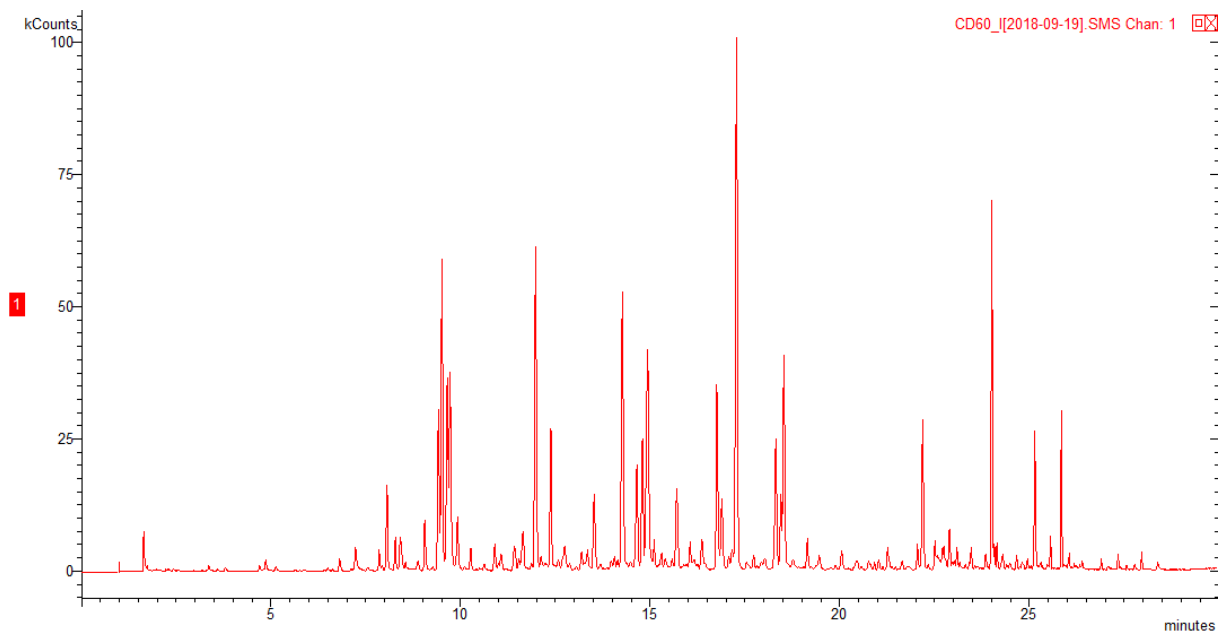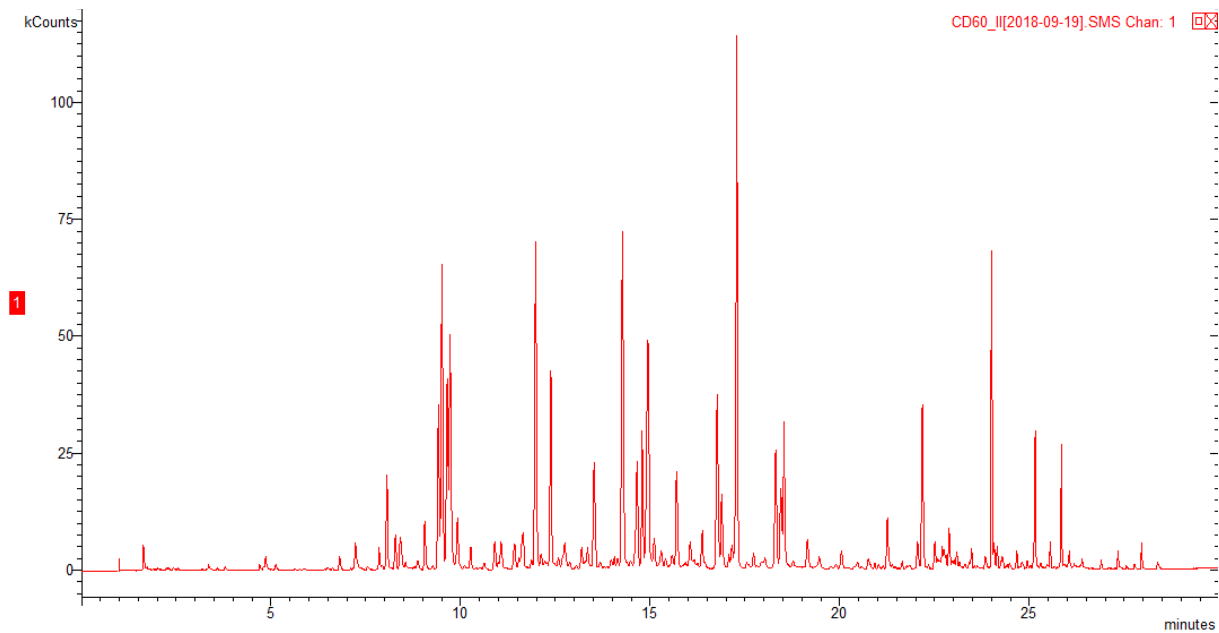

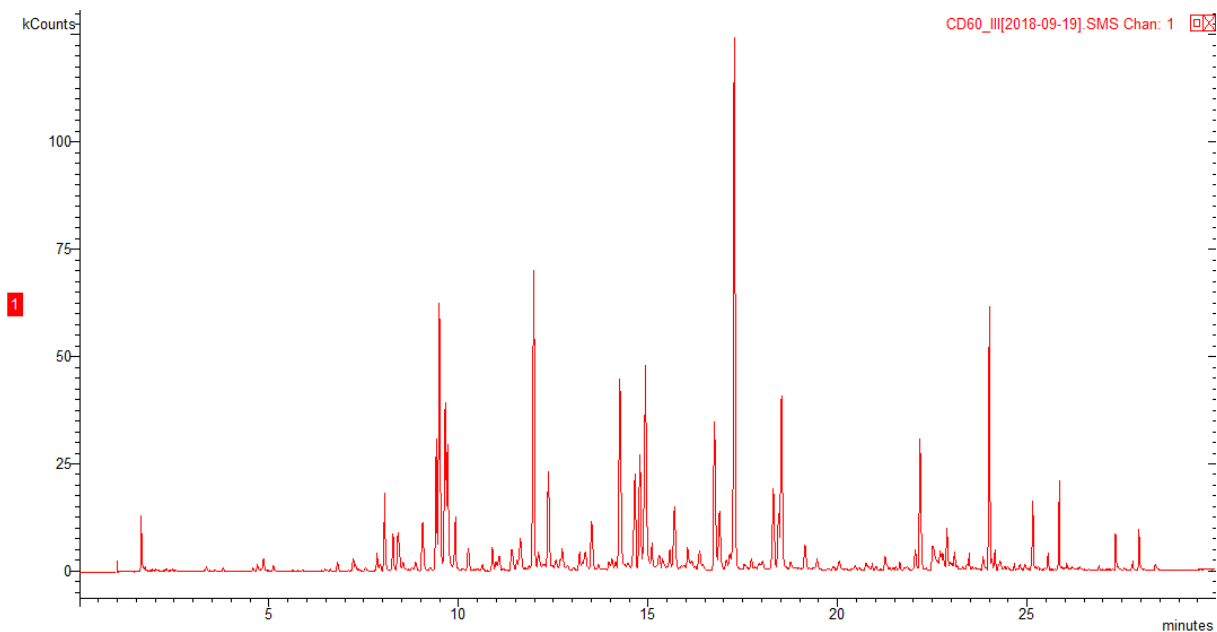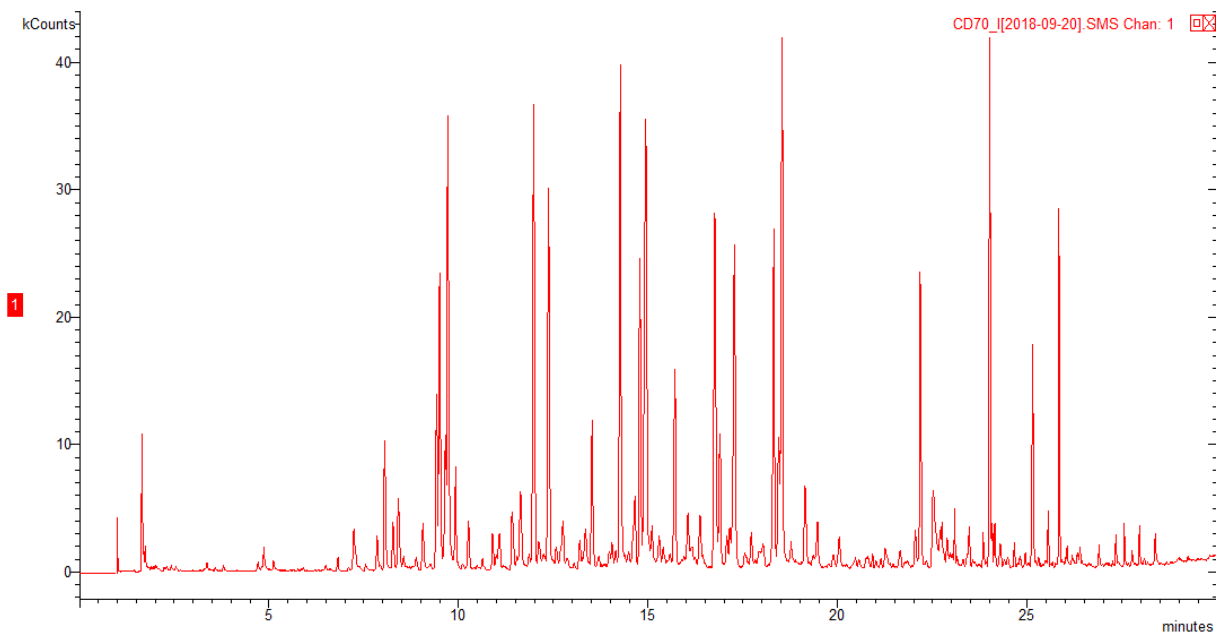

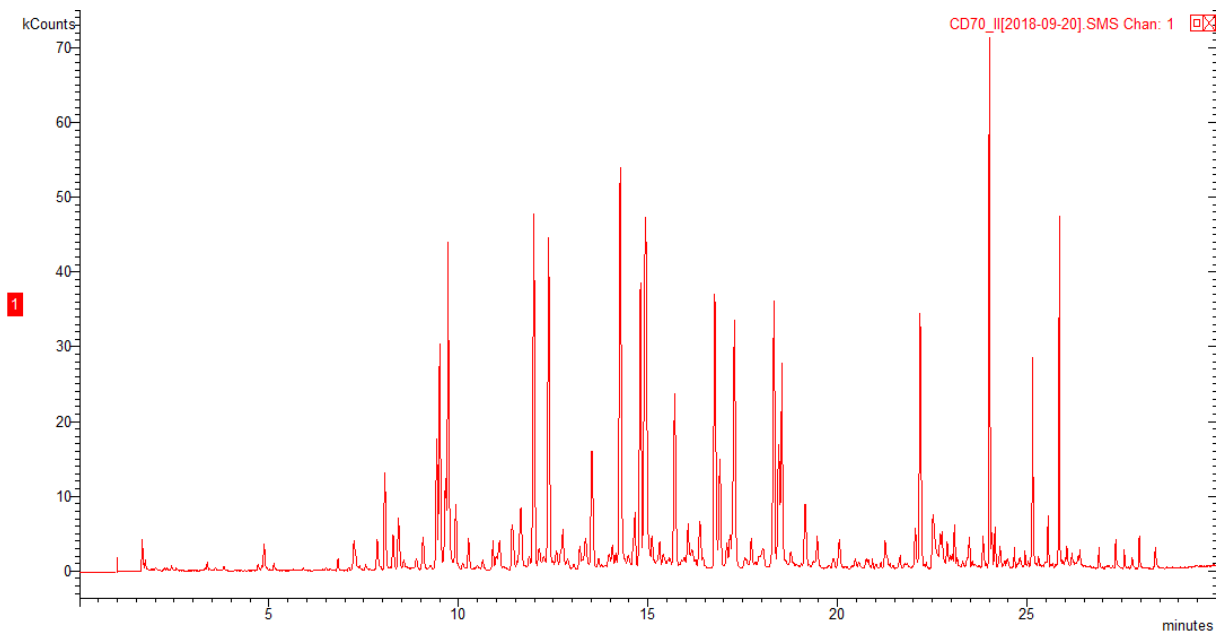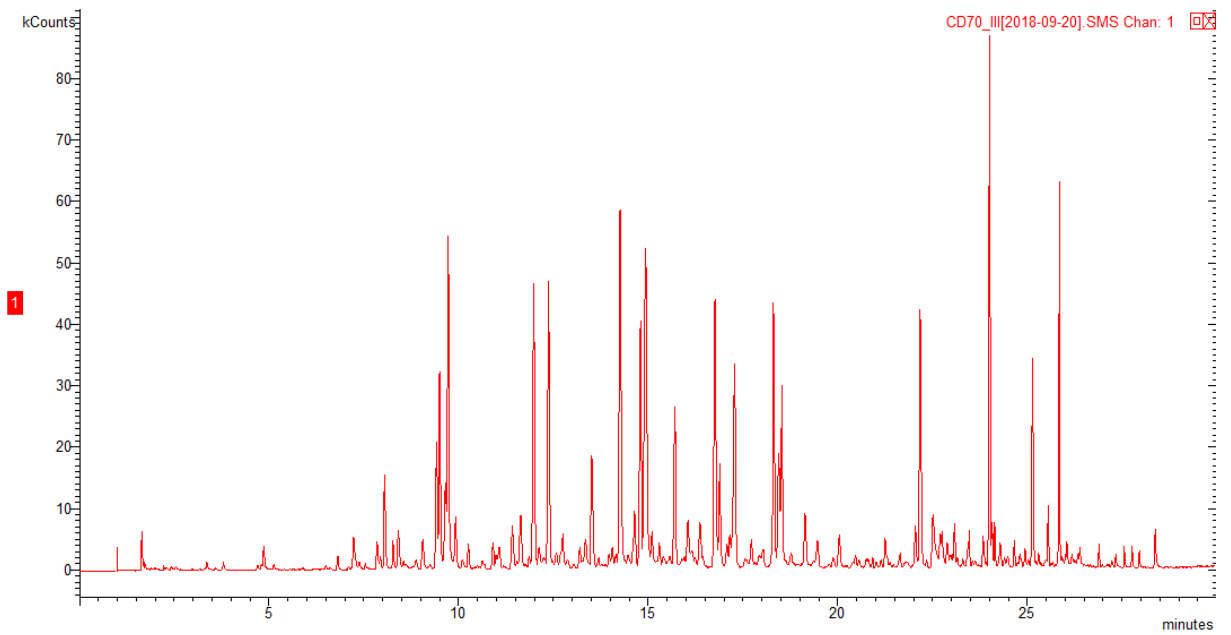

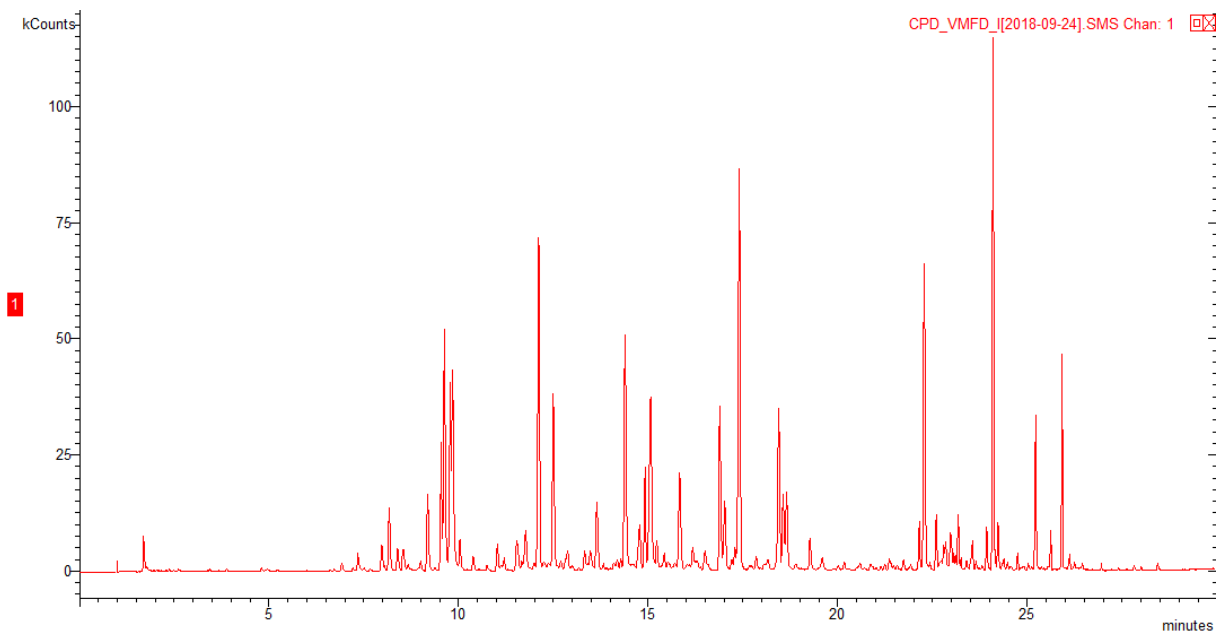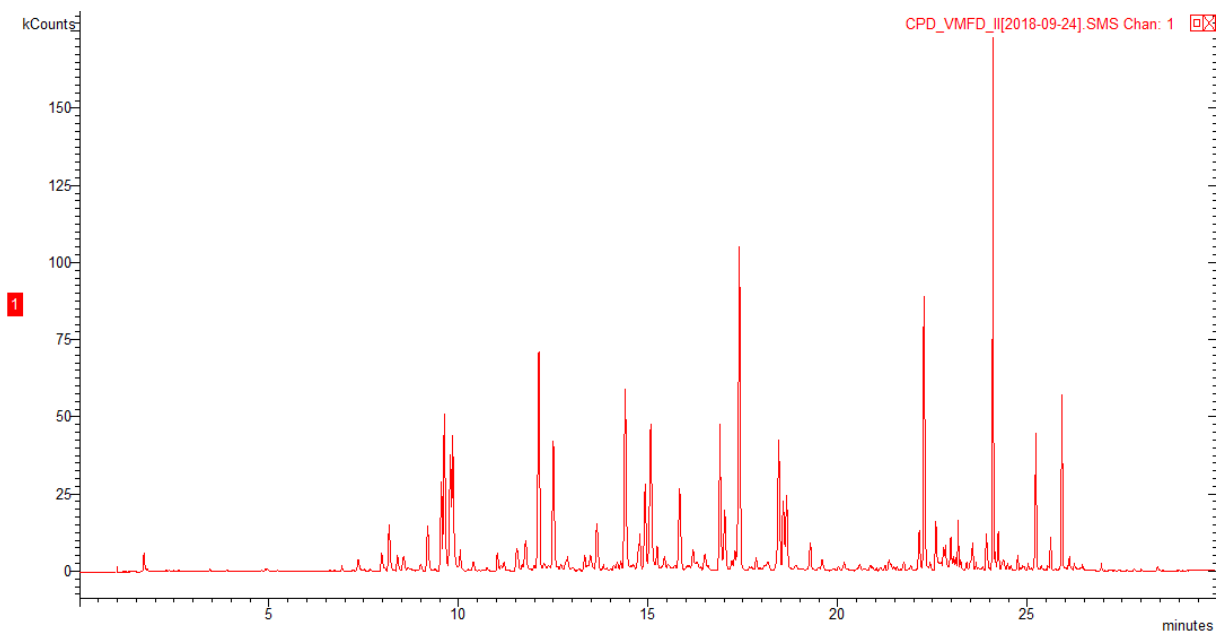

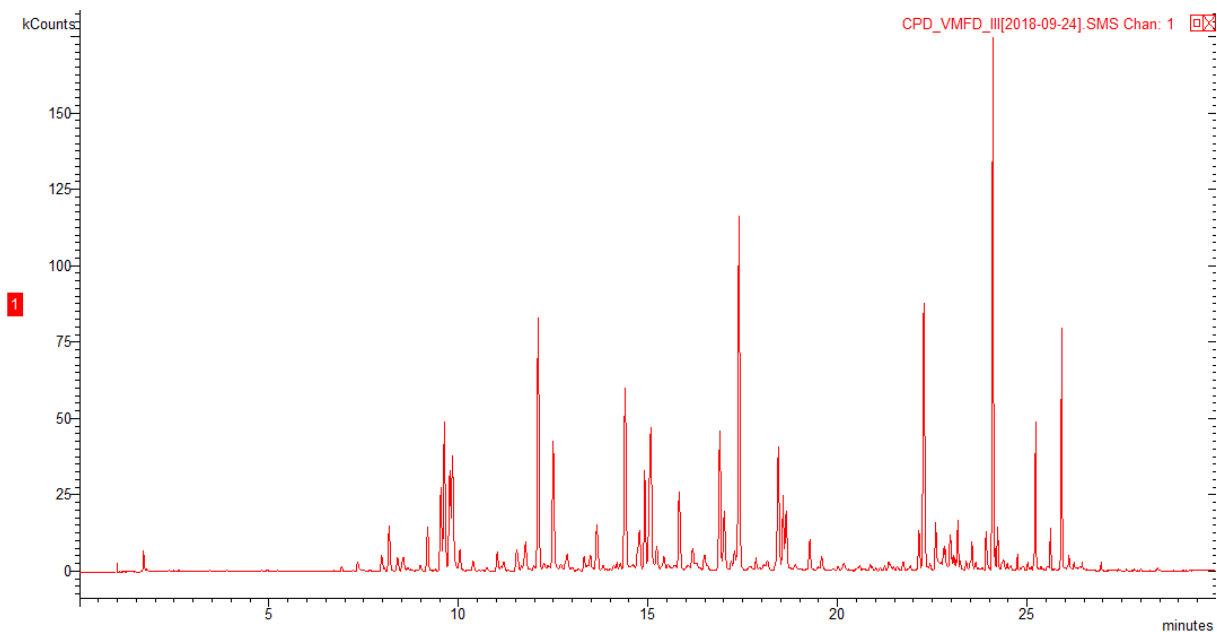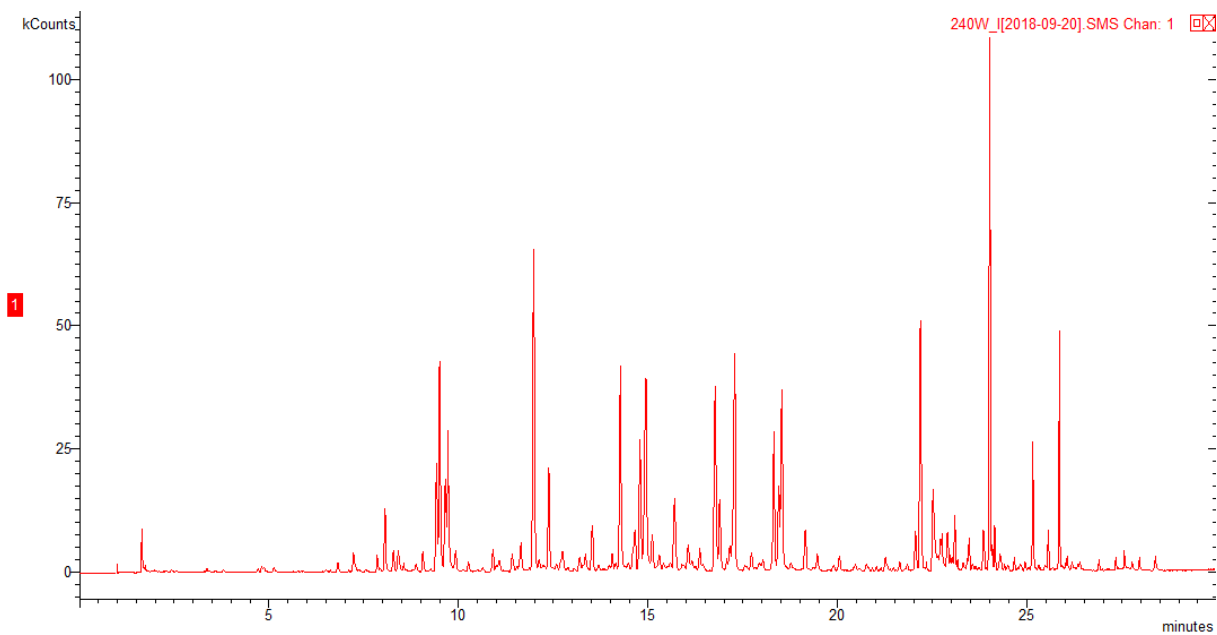

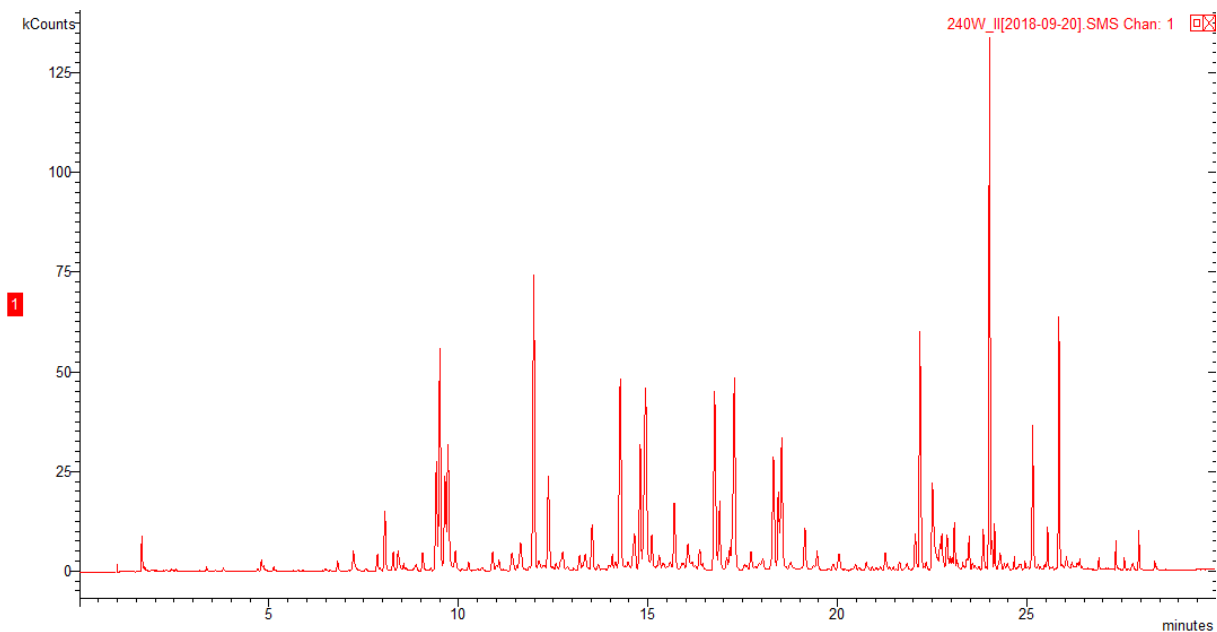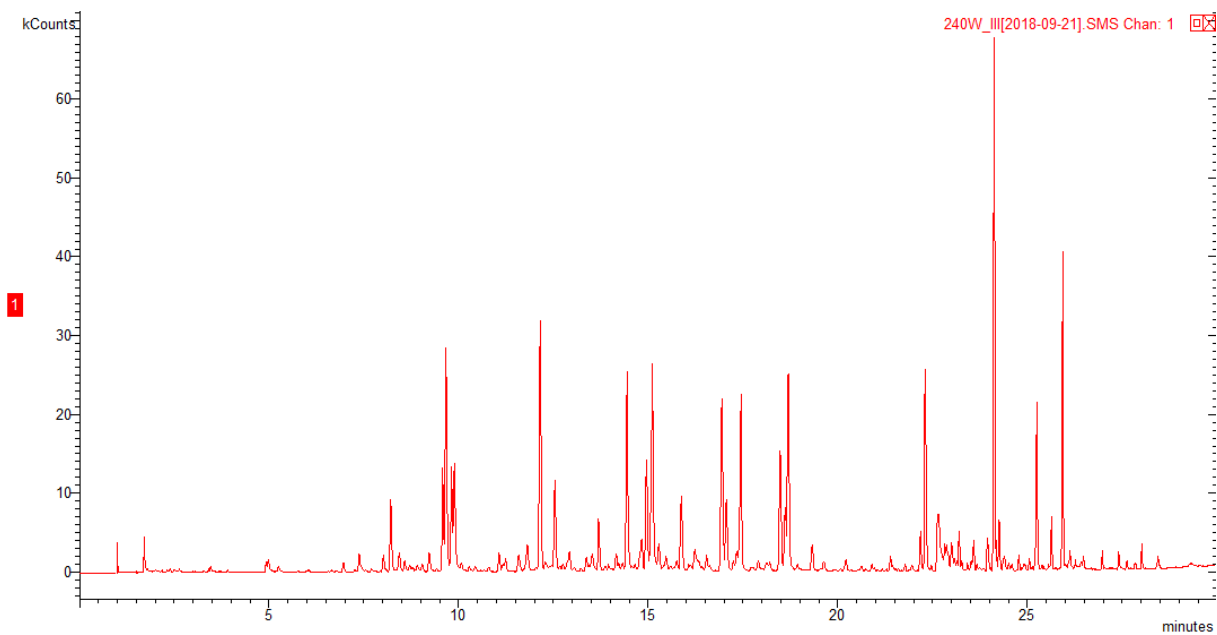

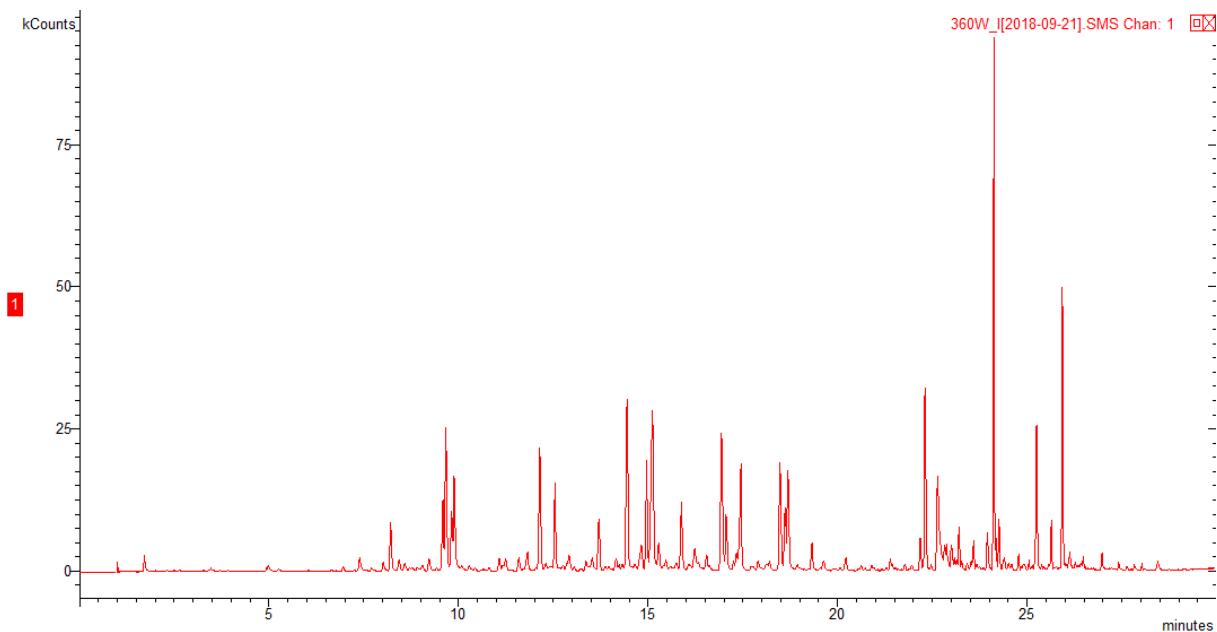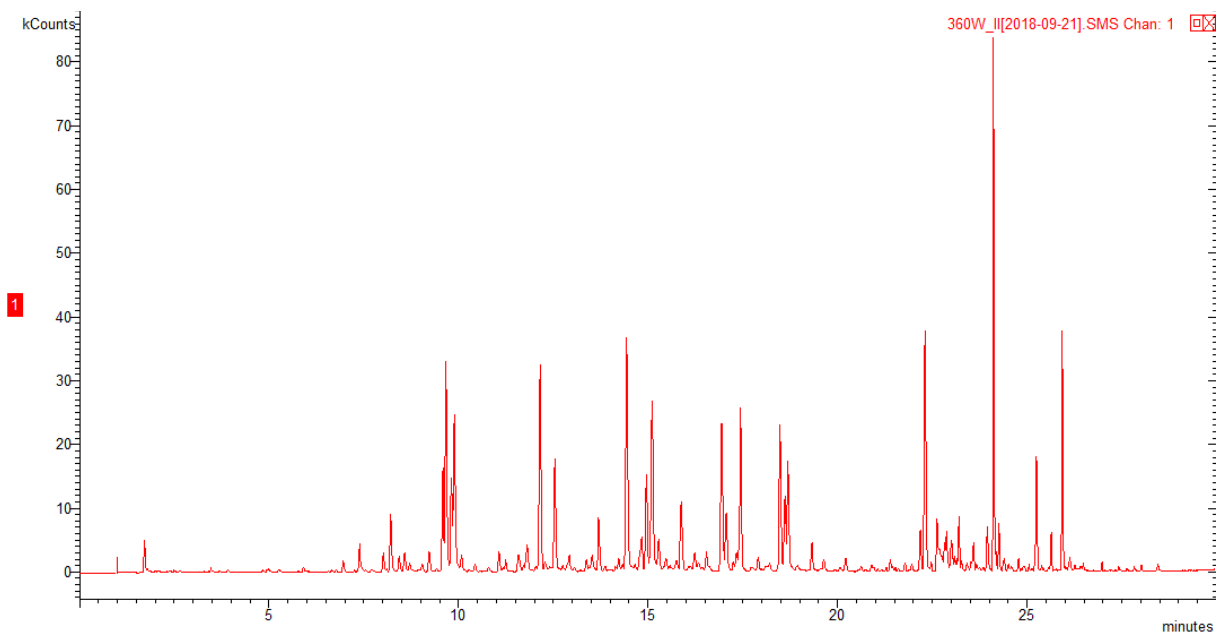

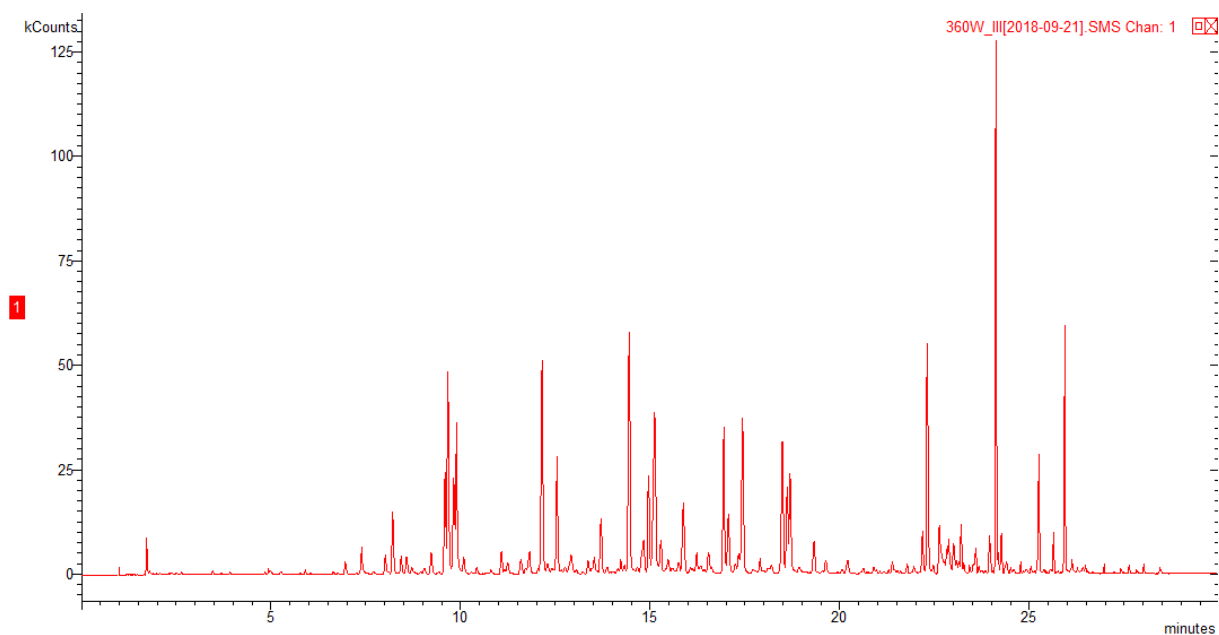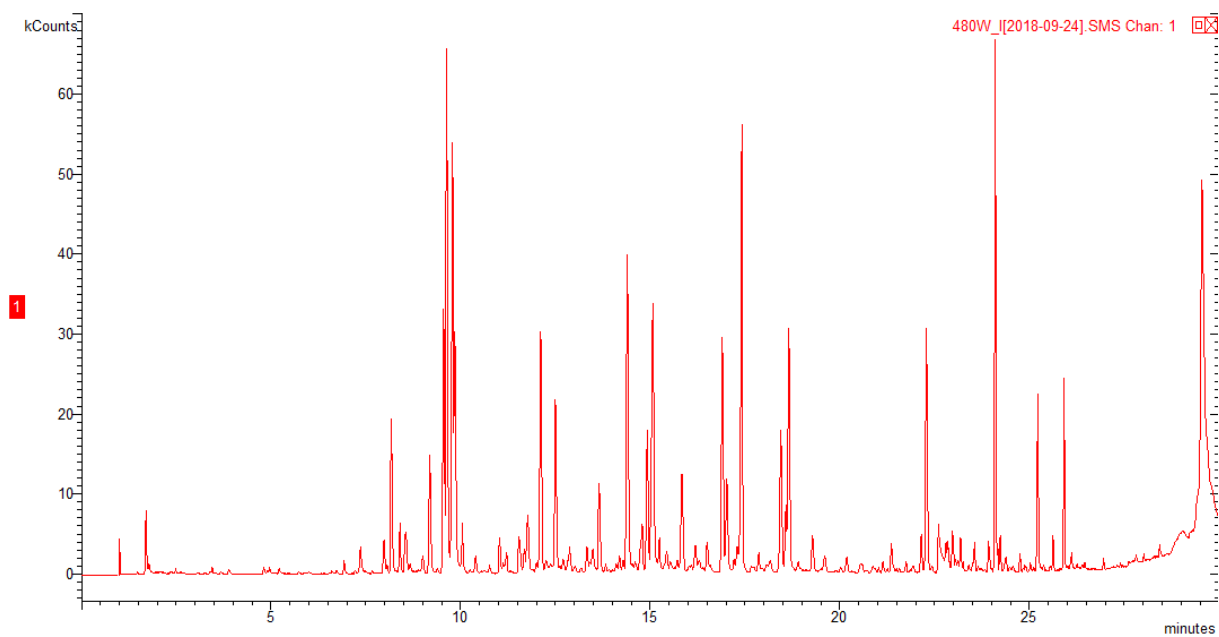

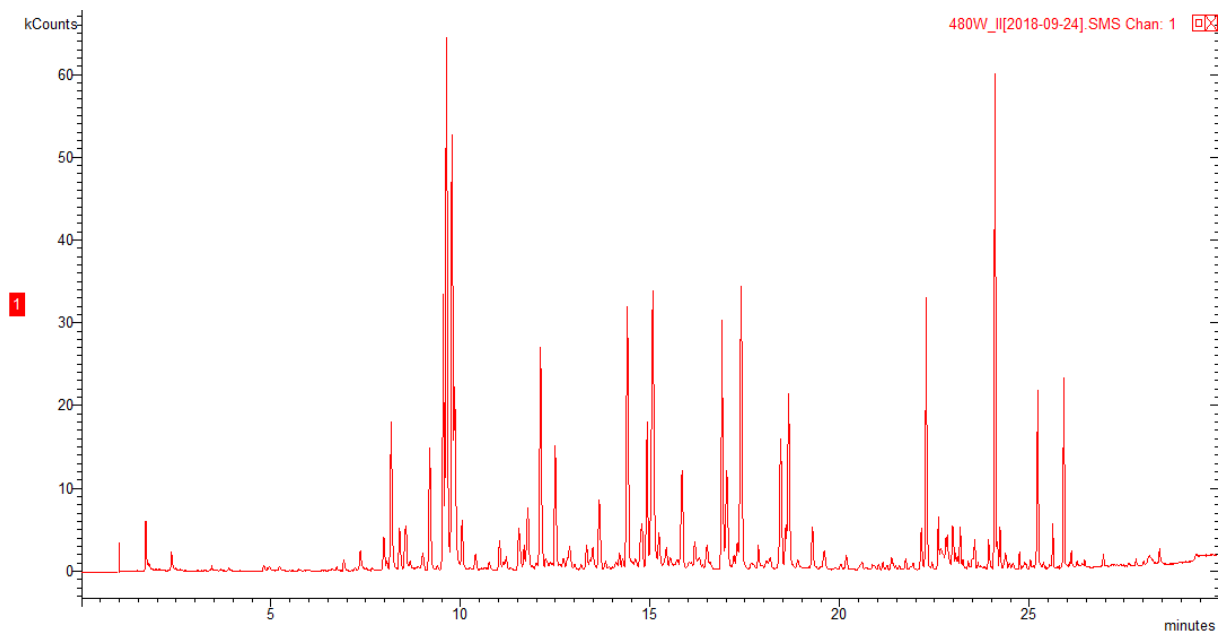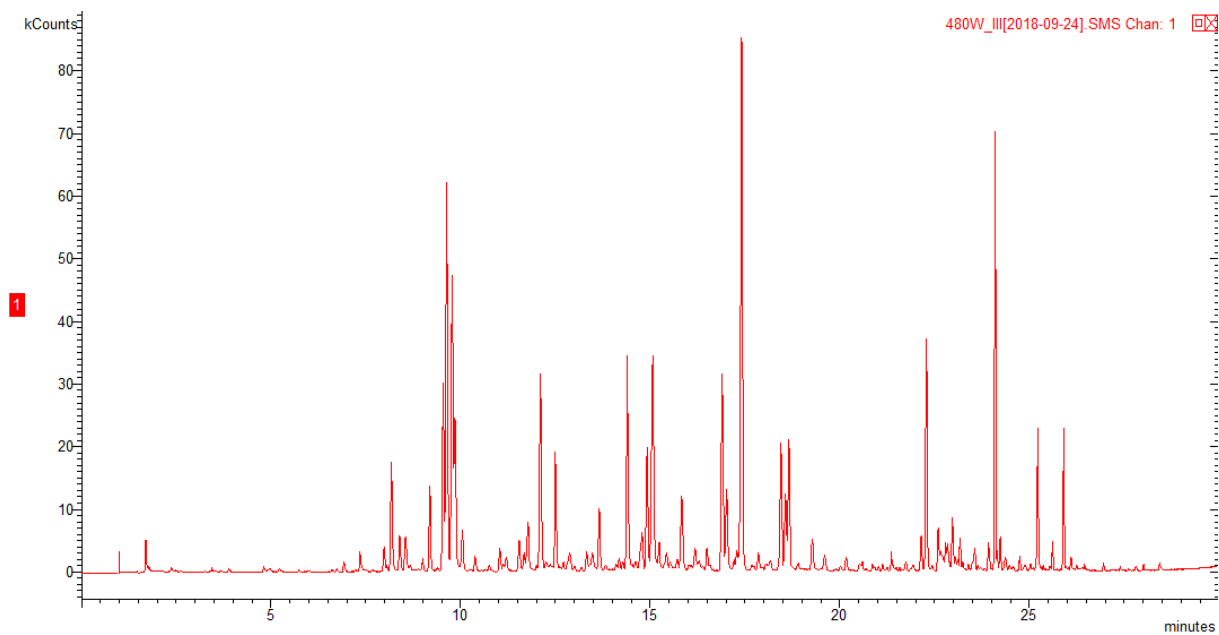

Supplement: Supplementary file 1 [file molecules-24-00764-s001.zip › Chromatograms.pdf]

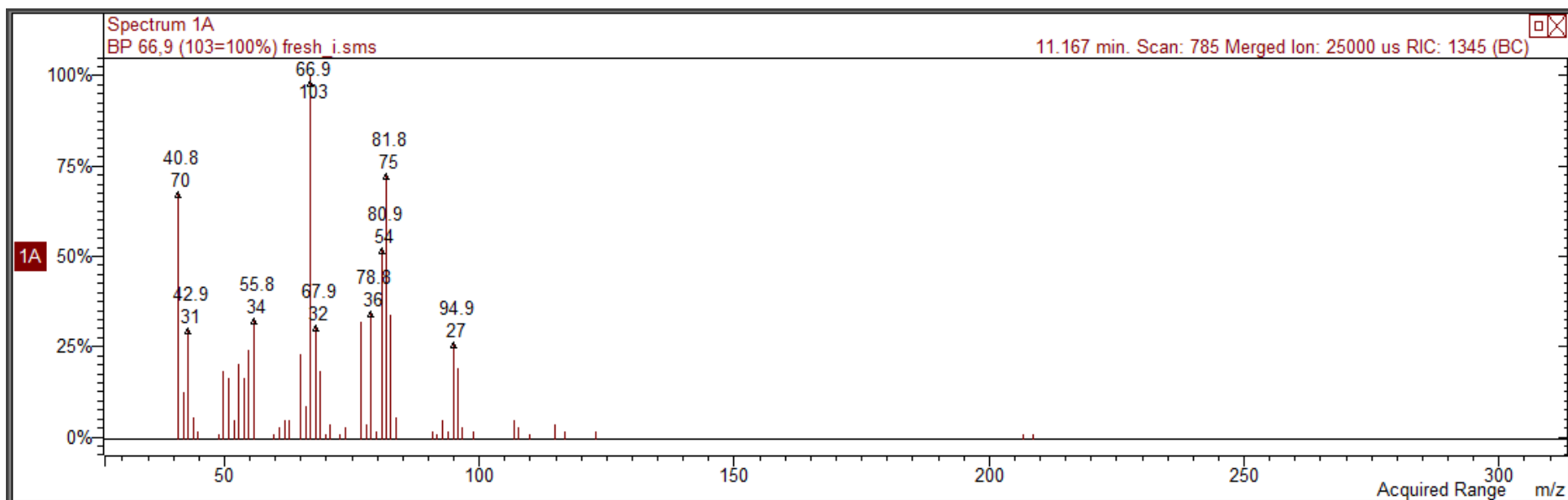

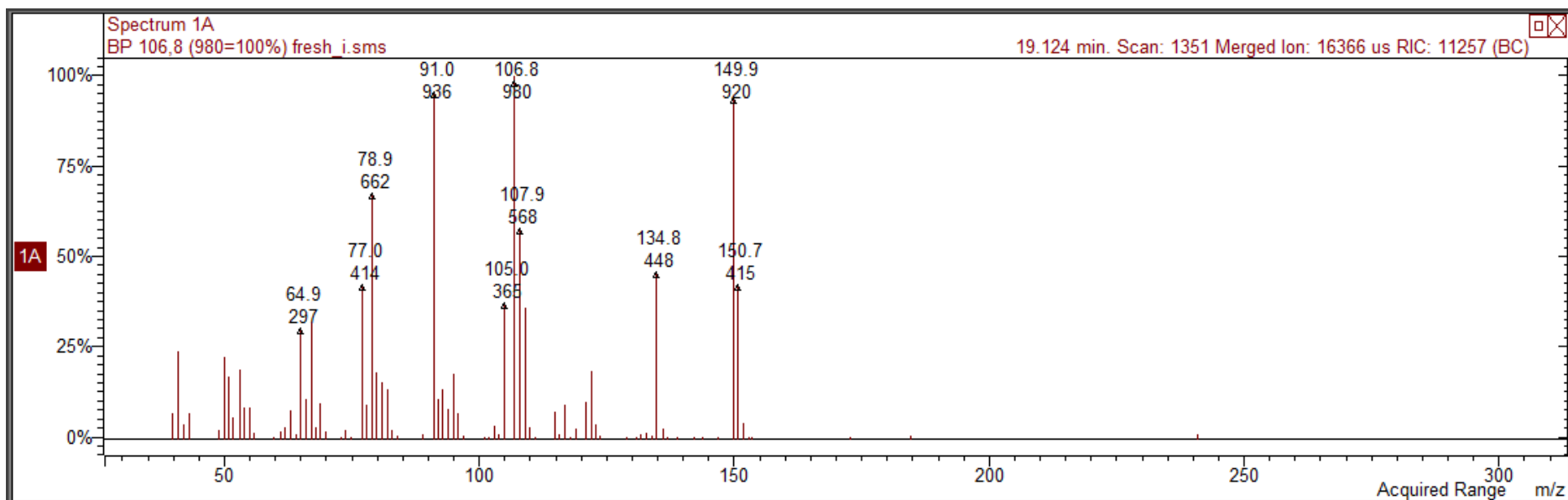

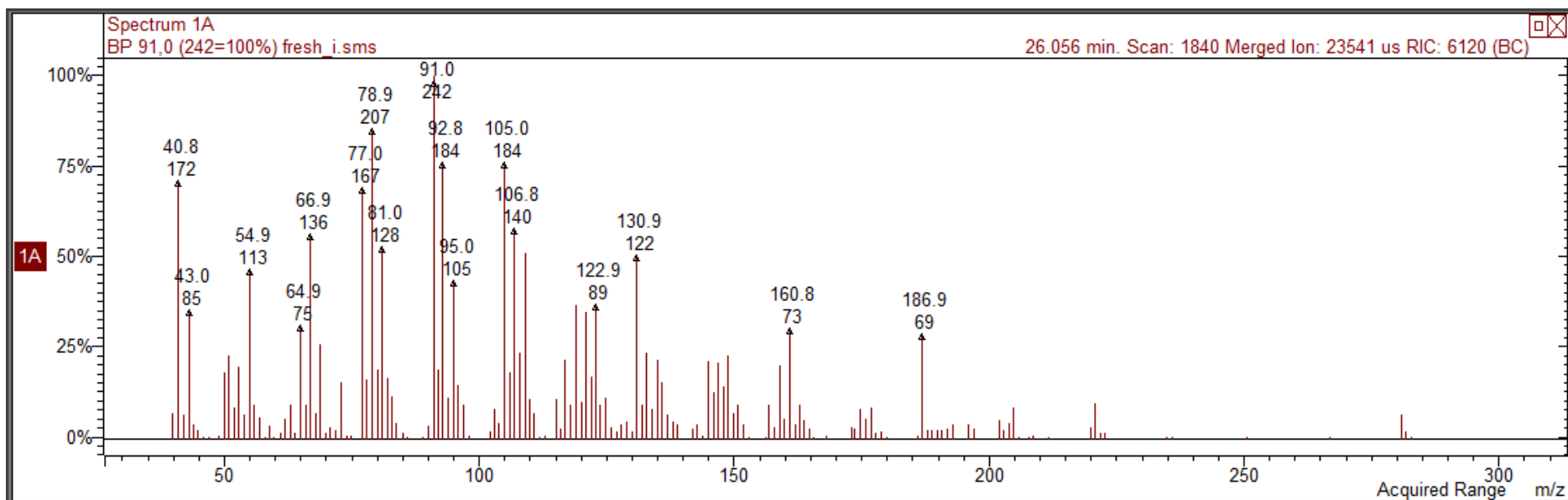

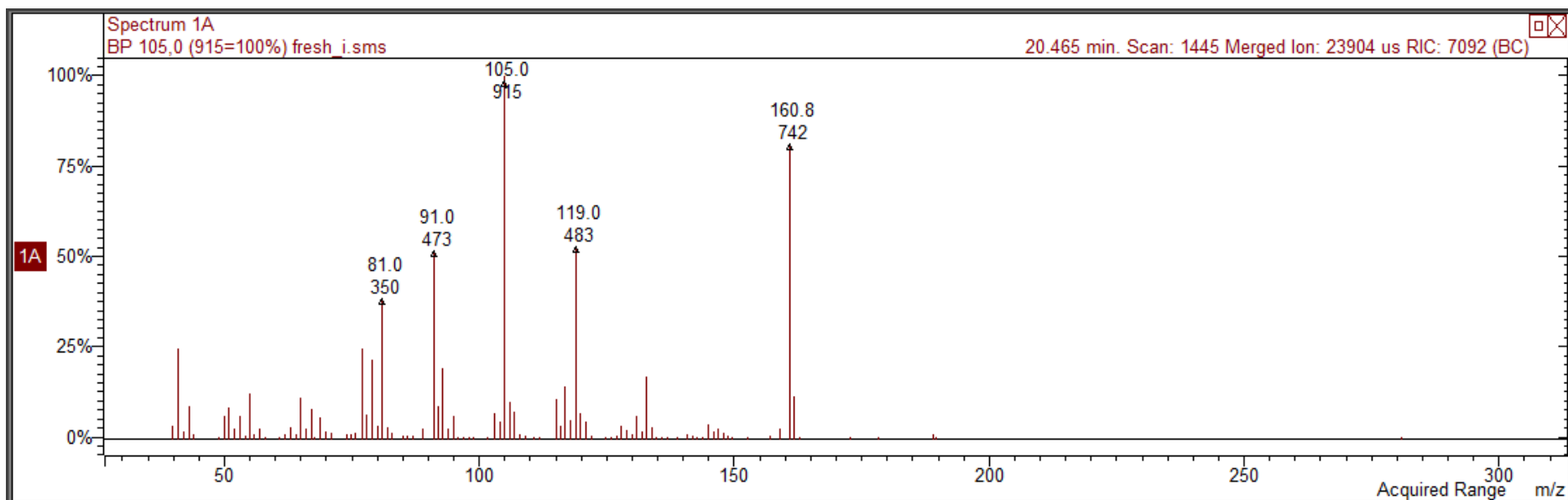

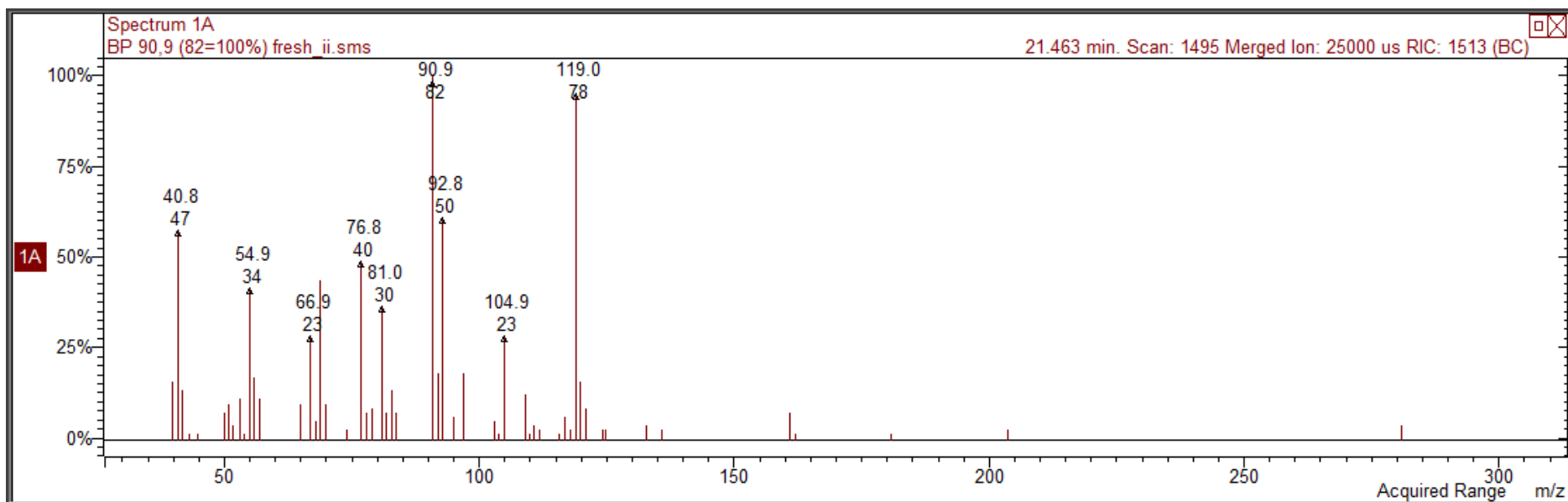

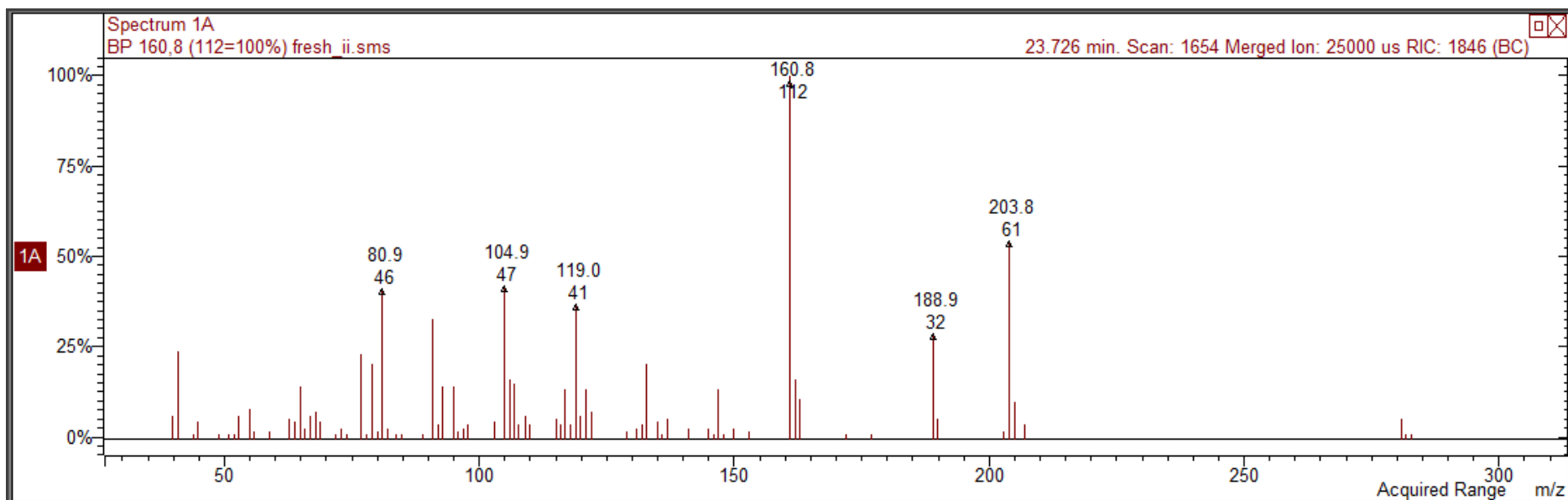

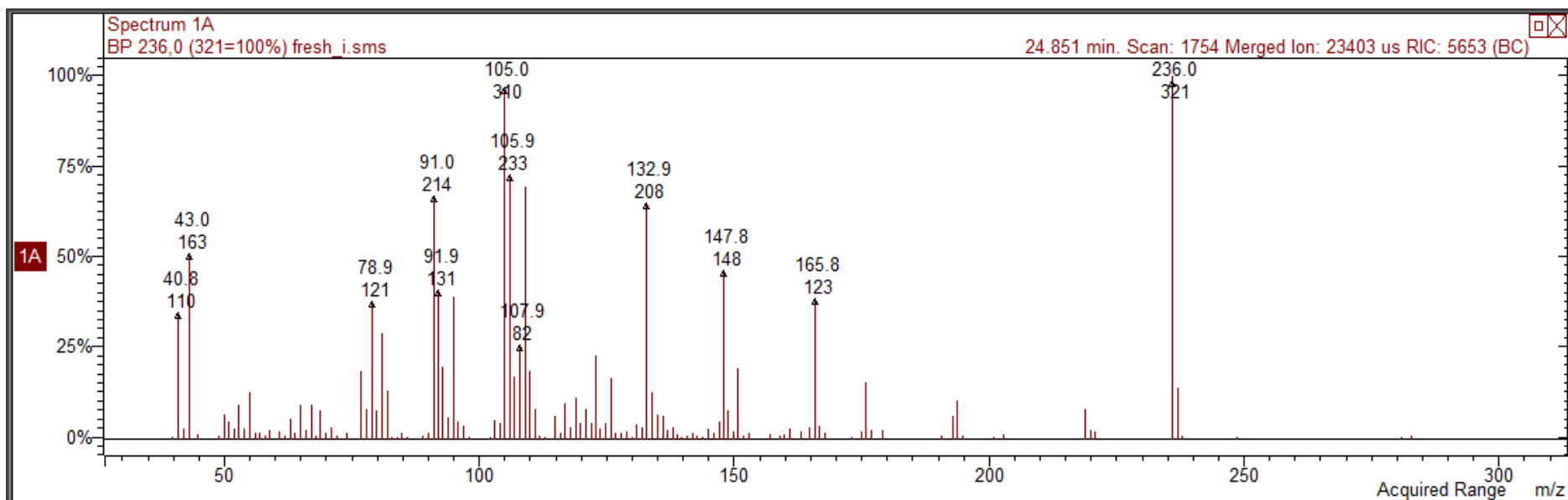

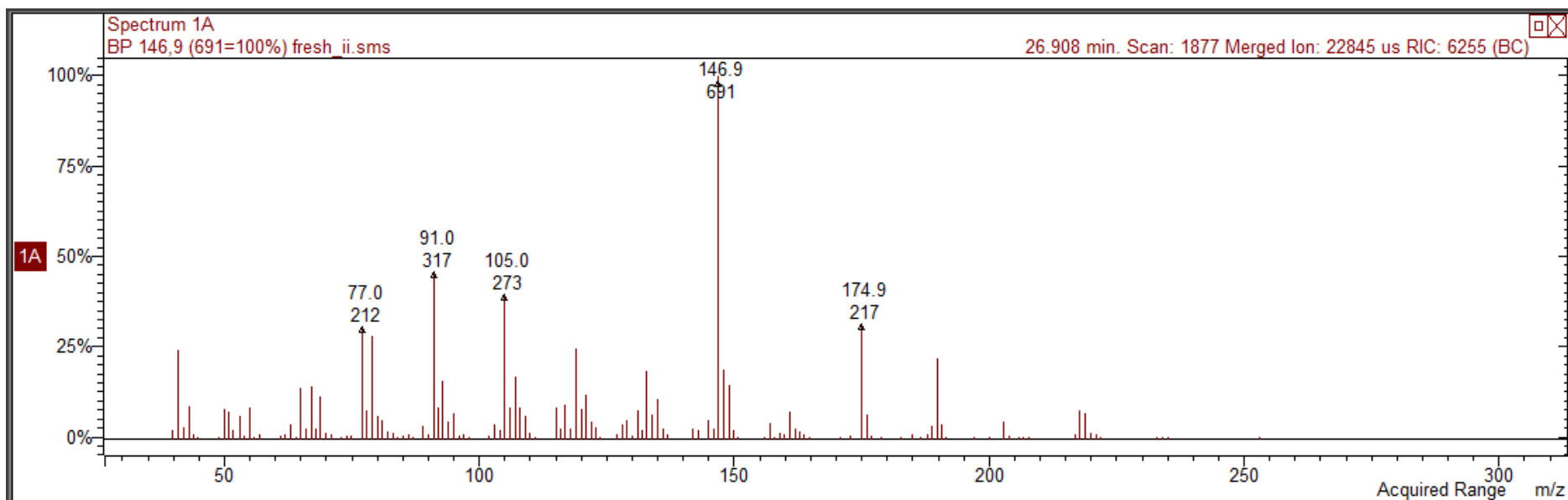

Supplement: Supplementary file 1 [file molecules-24-00764-s001.zip › unknown_compounds_mass_spectra.pdf]
